# Surface topography and spectrophotometric assessment of white spot lesions restored with nano-hydroxyapatite-containing universal adhesive resin: an in-vitro study

**DOI:** 10.1186/s12903-023-03642-3

**Published:** 2023-11-22

**Authors:** Neven S. Aref, Rahaf M. Alsdrani

**Affiliations:** 1 Dental Biomaterials Department, Faculty of Dentistry, Mansoura University, Mansoura, Egypt; 2 Basic Oral and Medical Sciences Department, College of Dentistry, Qassim University, Buraydah, Saudi Arabia; 3 College of Dentistry, Qassim University, Buraydah, Saudi Arabia

**Keywords:** Dentistry, Microanalysis, Remineralization, Nano-hydroxyapatite, Universal adhesive, White spot lesions

## Abstract

**Background:**

White spot lesion (WSL) is a main shortcoming accompanied by orthodontic treatment. It impairs the esthetic, surface hardness, and surface texture of enamel. So, this study was conducted to analyze the surface characteristics and color change of white spot lesions treated with nano-hydroxyapatite (nHA)-enriched universal adhesive resin.

**Materials and methods:**

Eighty sound human permanent molars crowns were sectioned into two halves, producing 160 specimens. 16 specimens were left untreated, and 144 specimens were artificially-demineralized to generate WSLs. The specimens were classified according to the treatment approach applied as follows: I; Sound enamel, Group II; artificially-created WSLs, Group III; ICON resin-restored WSLs, Group IV; Universal adhesive resin-restored WSLs, Group V; 0.5 wt% nHA-containing universal adhesive resin-restored WSLs, Group VI; 1 wt% nHA-containing universal adhesive resin- restored WSLs, and Group VII; 3 wt% nHA-containing universal adhesive resin-restored WSLs, Group VIII; 5 wt% nHA-containing universal adhesive resin-restored WSLs, Group IX; 7 wt% nHA-containing universal adhesive resin-restored WSLs, and Group X; 10 wt% nHA-containing universal adhesive resin-restored WSLs. Some surface characteristics and color changes were assessed. Data was collected and analyzed statistically using ANOVA and the Tukey test at *p* < 0.05.

**Results:**

Surface microhardness of WSLs was significantly improved with all investigated ratios of nHA-containing universal adhesive (*p* < 0.0001), with the highest mean belonging to 10 wt% nHA-containing universal adhesive resin treated WSLs. All ratios of nHA-containing universal adhesive resin significantly reduced the surface roughness of WSLs (*p* < 0.0001). The investigated ratios of 1, 3, 5, 7, and 10 wt% nHA-containing universal adhesive resin treatment approach could mask the WSLs significantly (*p* < 0.0001).

**Conclusions:**

Nano-hydroxyapatite-containing universal adhesive is a promising contemporary approach for the management of WSLs, coupled both the remineralizing concept and the minimally invasive resin infiltration.

## Introduction

Decalcification of the enamel, or white spot lesion (WSL), is typically brought on by the ongoing accumulation of bacterial plaque on the teeth. White spot lesions are caused by mineral loss in the surface or subsurface enamel and have a white appearance as a result of different optical reflections [1]. The prolonged interaction of the teeth’s outer surfaces with the acid created by bacterial metabolism in the biofilm may be caused by a number of initiating factors of both endogenous and exogenous origin. These factors include an acidic salivary pH, a diet high in acidic foods and low in calcium and phosphates, gastrointestinal tract disorders (such as gastroesophageal reflux disease and gastritis), eating disorders linked to vomiting, such as bulimia, a lack of oral hygiene, and orthodontic multi-bracket appliance therapy [2].

Enamel demineralization is of high incidence and can be very rapid in orthodontic patients compared to others. The consequences of WSLs may vary according to several factors, including plaque retention, particularly on the gingival side of orthodontic appliances, inadequate dental hygiene on the part of the patient, and the patient’s innate immunity [3].

White spot lesions’ management should include strategies for both stopping demineralization and promoting remineralization of existing lesions [4]. Correct oral hygiene practices and the use of remineralizing products based on calcium, phosphates, and fluorides are both necessary for dental enamel management [3]. Historically, fluoride has been the first attempt in dental practice to be used for preventive purposes [5]. Recently, casein phosphopeptide-amorphous calcium phosphate [6] and biomimetic hydroxyapatite have been introduced and showed promising results in this regard [7]. Therefore, recent research has shifted toward the development of innovative remineralizing methods that are based on the integration of calcium and phosphates at the level of demineralized dental surfaces as an alternative to fluoride [8].

The use of nanoparticles in restorative and preventive dentistry has been investigated by certain researchers since the development of nanotechnology [9–11]. Because of its resemblance to the bone and mineral structure of teeth, biocompatibility, and bioactivity, nano-hydroxyapatite (nHA) was viewed as promising [12]. Preceding studies [13, 14] have proved that nano-hydroxyapatite, when added to mouthwashes and toothpastes, can remineralize artificial carious lesions. Nano-HA toothpastes outperformed amine fluoride toothpastes in terms of dentinal remineralization [15].

Resin infiltration is a recent microinvasive method of treating white spot lesions. In this procedure, there is a high penetration of a low-viscosity resin to fill the demineralized areas and stop further demineralization, and acceptable aesthetic results are shown by short-term trials. The demineralized enamel’s inter-crystalline gaps and pores are filled with resin, which forms a diffusion barrier on the surface and in the enamel’s deeper layers. This prevents acids from entering the enamel, and slowing the advancement of the lesions. According to recent studies, resin injection can bring WSLs’ color back to a level that is clinically acceptable [16–19]. When compared to both remineralization alone and the routine application of fluoride varnishes, resin infiltration greatly improved WSLs optically more than both of these methods [20]. Fluoride varnish only ever offered optical outcomes that were comparable to resin infiltration in one investigation; however, this optical improvement required up to 6 months after fluoride treatment, whereas a subsequent improvement was visible following resin infiltration [21].

Resin infiltration had also been tried as a subsequent approach after bleaching stained enamel. When the stain is completely eradicated after bleaching, it can be stated that external bleaching combined with the resin infiltration approach offers promising results for aesthetically managing stained enamel opacities [22]. Another investigation claimed that whether or not a bleaching procedure was applied, the resin infiltration increased the WSLs’ microhardness, while bleaching procedures prior to resin infiltration caused color changes and were discovered to have a detrimental effect on the infiltrant’s ability to penetrate [23]. Furthermore, the resin infiltration method has been identified as the most effective and promising strategy for the management of dental fluorosis. This complies with the idea of minimally invasive restorative dentistry and enables the achievement of satisfying outcomes without the need for unnecessary tissue removal [24]. The recently introduced adhesives are universal adhesives, which contain 10-methacryloyloxydecyl dihydrogen phosphate (MDP), an acidic functional monomer used in these adhesives to produce surface micro-retention and chemical interactions with calcium found in tooth hydroxyapatite. The MDP-Ca water-insoluble salts protect the collagen fibers from deterioration [ 25]; this concept may be considered in further studies with long-term follow-ups to confirm its influence on the stability of the esthetic results based on this kind of adhesive in the management of WSLs.

Despite the fact that the remineralizing agents can aid in enamel remineralization, the aesthetic is still greatly impaired [26, 27]. The research hypothesis here is based on two main principles: the universal adhesive resin (esthetic-restoring approach) will provide efficient resin infiltration into demineralized enamel, reducing the refractive index of the microporosities and enhancing aesthetics. Furthermore, due to its unique structure, it will be able to chemically bond with the hydroxyapatite of the tooth structure as well as the nano-hydroxyapatite used in the treatment of WSLs. On the other hand, nano-hydroxyapatite (the remineralizing approach), by virtue of its exclusive and evidenced remineralizing ability, will be able to restore the artificially-created WSLs. Another advantage of this strategy is that it is not dependent on patient compliance, which may cause the treatment to be stopped before it is completed. So, the treatment of WSLs using nano-hydroxyapatite-containing universal adhesive resin as an alternative approach for restoring demineralized enamel could be an encouraging approach for regaining the surface hardness, texture, and esthetic of demineralized enamel. The null hypothesis was that this treatment approach would not be able to treat WSLs successfully, and esthetic may remain impaired. Surface microanalysis in terms of surface microhardness, surface roughness, and scanning electron microscopy was carried out. Additionally, spectrophotometric assessment of the treated WSLs was performed.

## Materials and methods

### Teeth selection and study design

**Fig. 1:**
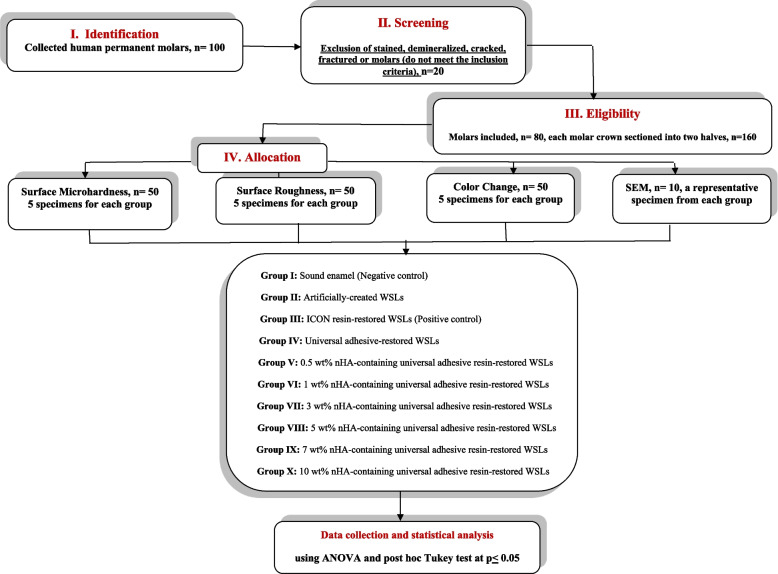
Flow diagram of the experimental design

The study was conducted in accordance with the ethical approval of the institutional scientific research committee at the College of Dentistry, Qassim University, Saudi Arabia (#EA/6096/2021). In the outpatient clinic at the College of Dentistry, Qassim University, extracted sound permanent molars were collected. The teeth were cleaned in distilled water to eliminate any traces of blood or soft tissue using a brush. The teeth were then placed in an aqueous solution containing 0.1% thymol. Dryness and careful inspection of the teeth by a stereomicroscope (Olympus, Japan, SZ-PT, 10x magnification) to exclude those with stains, demineralization, or enamel cracks. Using a microsaw (Isomet 4000, Buehler, USA), the roots of the carefully chosen teeth were cut at the cemento-enamel junction. The following step involved cutting the 80  crowns into 160 specimens by sectioning each one buccolingually into two segments. Following that, the specimens were randomly divided into 10 groups, as follows: Group I; Sound enamel specimens (Negative control), Group II; Artificially-created WSLs specimens, Group III; ICON resin-restored WSLs (Positive control), Group IV; Universal adhesive resin- restored WSLs, Group V; 0.5 wt% nHA-containing universal adhesive resin-restored WSLs, Group VI; 1 wt% nHA-containing universal adhesive resin- restored WSLs, and Group VII; 3 wt% nHA-containing universal adhesive resin-restored WSLs, Group VIII; 5 wt% nHA-containing universal adhesive resin-restored WSLs, Group IX; 7 wt% nHA-containing universal adhesive resin-restored WSLs, and Group X; 10 wt% nHA-containing universal adhesive resin-restored WSLs (Fig. 1: Flow chart of the experimental design).

### Enamel demineralization (artificial creation of WSLs)

First, coating the enamel surface with a double coat of colorless acid-resistant nail polish, leaving a round working window of 5 mm diameter, was done, then left in the air to dry for 24 h. Regarding the artificial creation of the white spot lesions in sound enamel, hydroxy ethyl cellulose (HEC) gel (pH 4.95–5) was used. The gel was prepared by dissolving the HEC powder (Sigma Aldrich co., Lot # BCCD1680, Germany) into distilled water in a ratio of 35 g/250 mL. A solution containing 0.05 M lactic acid (DL-Lactic acid, Sigma Aldrich co., Lot # BCCD0769, Germany) was used to adjust the gel acidity which was checked using a pH meter (pH -2011, LIDA, China). Stirring the mixture for 30 min for partial hydrolysis, then incubating at 37 °C for 24 h to ensure complete hydrolysis. The demineralizing gel was then added to the container containing encompassing the specimens representing each group, ensuring that they remained submerged for 10 consecutive days with three renewals. After completion of the demineralization process according to the assigned protocol, specimens were removed from the gel, washed with deionized water for 20 sec, and air-dried for 10 sec. Only specimens of group I (Sound enamel) left without demineralization.

### Preparation of the nano-hydroxyapatite/universal adhesive resin different formulations

Hydroxyapatite nano-powder (Sigma Aldrich co., Lot # MKCK8172, Germany, particle size < 200 nm as received by the manufacturer) was mixed with the light-curing self-etching universal adhesive resin (GLUMA Bond Universal, Lot # K010039, Heraeus Kulzer), in different ratios of; 0.5 wt%, 1 wt%, 3 wt%, 5 wt%, 7 wt%, and 10 wt%. The nHA powder and the resin liquid were weighed using a four-digit, precise balance. The considered proportions of each assigned ratio were mixed in dark small glass bottles and kept on a magnetic stirrer for 24 h to ensure a homogenous mix.

### Treatment of the demineralized specimens

Specimens of groups III, IV, V, VI, VII, VIII, IX, and X were treated with the specified protocols of each group, following the manufacturer’s recommendations concerning light intensity and curing time.

The ICON resin infiltrant (DMG Dental Materials, Hamburg, Germany) was applied to specimens of group III in accordance with the manufacturer’s guidelines. The experimental surfaces received a direct application of ICON etchant containing 15% HCL, which was left on for 2 min, and then washed off with water for 30 sec. ICON dry (99% ethanol) was applied to the working area, which was then allowed to air dry for 10 sec after the area had been dehydrated for 10 sec with an air-water spray. The WSLs were coated with ICON resin infiltrant using the resin applicator before being light-cured after 3 min of air drying. For groups IV, V, VI, VII, VIII, IX, and X, the nHA-containing universal adhesive resin was applied and cured. All specimens were coated into two coats, and cured for 40 sec/each coat with the LED light curing unit (Elipar S10, 3 M ESPE, USA) of 1200 mW/cm^2^ intensity. Specimens of all groups were stored in deionized water at 37 °C for 48 days before testing.

### Scanning Electron microscopy

The surface of a symbolic specimen from each experimental group was microscanned (magnification × 2000) to detect the topographical changes within the enamel surface after being treated. Each specimen was gold plated using a sputter coating machine (Emitech-K500X, Quorum Technologies, Ashford, UK) before being inspected by means of a scanning electron microscope (JEOL, JSM-6510LV, Japan).

### Surface microhardness

Surface microhardness was evaluated using a Vickers microhardness tester (Micromet II, Buehler, Lake Bluff, IL, USA). The indenter was pushed into the polished test specimen with a constant, impact-free force equivalent to a mass of 50 g, and it was left in situ for 10 sec. The physical quality of the indenter and the accuracy of the applied load have to be under control to provide constant, precise results. The indentation was focused using the magnifying eyepiece once the load had been removed, and the two impression diagonals were then measured with a filar micrometer. For each specimen, this process was carried out three times, with the average value being taken into account. The Vickers hardness number (VHN) was calculated and stated in kgf/mm^2^.

### Surface roughness

A surface profilometer (Surftest 211, Mitutoyo, Tokyo, Japan) was used to measure the surface roughness. Device calibration was done using the standard calibration specimen before use. The following testing parameters were used: measuring distance (6 mm), speed (0.5 mm/s), and force (0.75 mN). Regarding the stylus profile, the tip radius was 2 μm and the tip angle was 60^°^. The surface roughness evaluation parameter (Ra) of each specimen was investigated in five different locations, and the average was calculated and expressed in μm.

### Spectrophotometric assessment (color changes)

The color difference of the experimental specimens was evaluated using a spectrophotometer (Vita Zahnfabrik H. Rauter GmbH & Co. KG, Bad Sackingen, Germany). CIE L*a*b* color parameters were used to express readings from spectrophotometers. The following formula was used to calculate the total color difference (ΔE) of the treated specimens with respect to sound enamel as a baseline:

$$\boldsymbol{\varDelta} \boldsymbol{E}=\left[\left(\boldsymbol{\varDelta} \boldsymbol{L}\ast \right)\ ^\textbf{2}+\left(\boldsymbol{\varDelta} \boldsymbol{a}\ast \right)\ ^\textbf{2}+\left(\boldsymbol{\varDelta} \boldsymbol{b}\ast \right)\ ^\textbf{2}\right]\ ^{\textbf{1}/\textbf{2}}$$

where a* and b* denote chromaticity and L* the color value. It was deemed that a clinically satisfactory color change occurred when the ΔE < 3.3.

 Power Analysis and Sample Size Software (version 16, 2018) (PASS, NCSS, LLC. Utah, USA) was used to determine the sample size. A total of 160 specimens were required to provide 16 specimens per group. The design achieves 95% power when an F test is used at a 5% level of significance and the effect size is 0.4. Shapiro-Wilk normality test was used to verify the normality of the data. Data analysis was conducted via the “Statistical Package for Social Sciences 25” (SPSS Inc., Chicago, IL, USA). Data were subjected to one-way variance analysis (ANOVA) and multi-comparison testing by Tukey post hoc at a level of significance, α = 0.05.

## Results

**Fig. 2:**
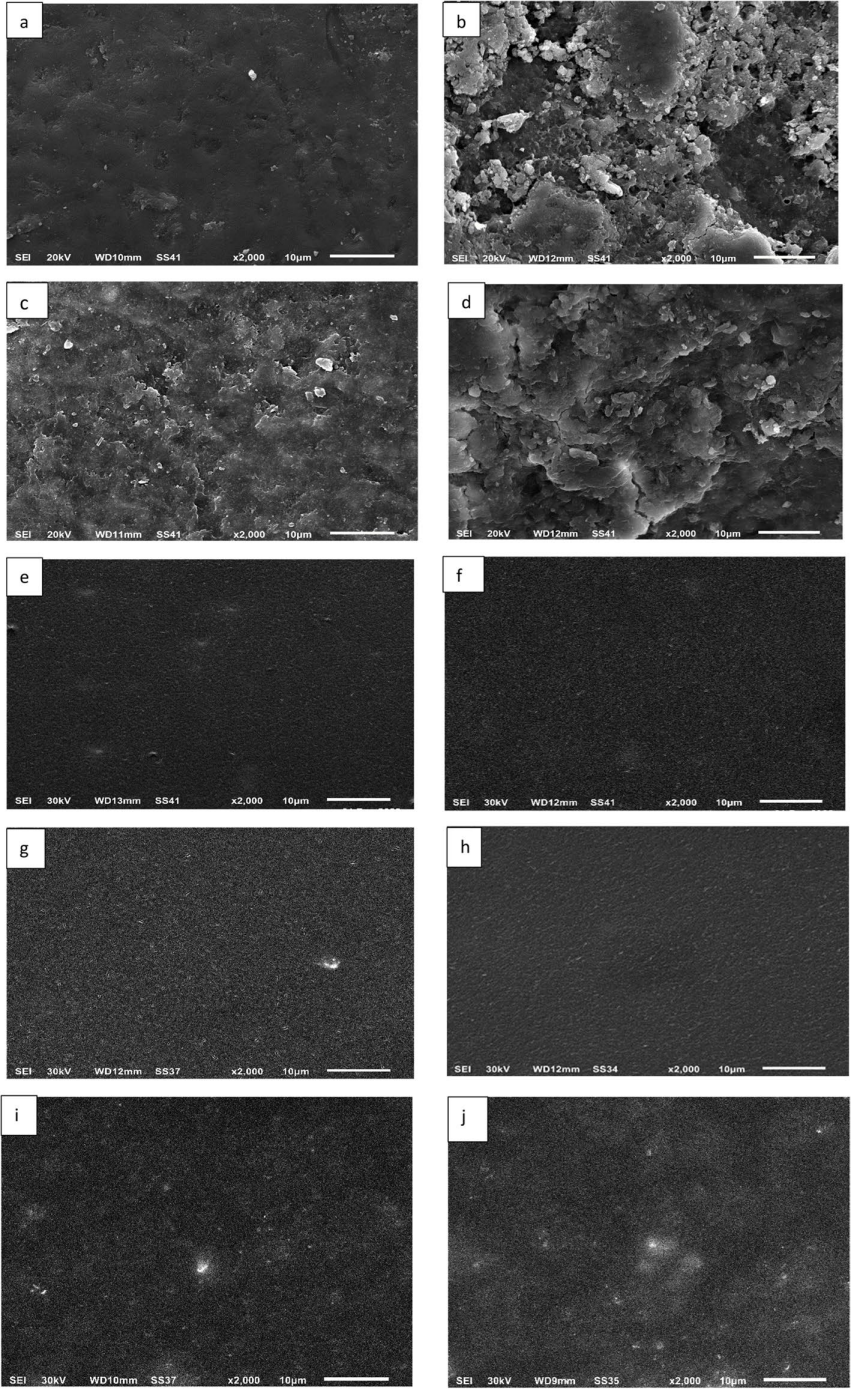
SE micrographs (× 2000 magnification) showing enamel surface of **a**, Control specimen (Sound enamel). **b**, Artificially-created white spot lesion (WSL). **c**, ICON resin-restored WSL. **d**, Universal adhesive resin-restored WSL. **e**, 0.5 wt% nano-hydroxyapatite (nHA)-containing universal adhesive resin-restored WSL. **f**, 1 wt% nHA-containing universal adhesive resin-restored WSL. **g**, 3 wt% nHA-containing universal adhesive resin-restored WSL. **h**, 5 wt% nHA-containing universal adhesive resin-restored WSL. **i**, 7 wt% nHA-containing universal adhesive resin-restored WSL. **j**, 10 wt% nHA-containing universal adhesive resin-restored WSL

Figure 2 represents the scanning electron micrographs of the representative specimens of the different experimental groups. Figure 2a, displays the sound enamel with a smooth, uninterrupted surface containing only some scattered pits and scratches with the characteristic fish scale appearance. Figure 2b, represents demineralized enamel, with the extremely irregular, pitted, and discontinued surface denoting the destruction of the enamel rods and the dissolution of the enamel minerals. Figure 2c and d, respectively, reveal the ICON resin and the universal adhesive resin, ability to frequently infiltrate the demineralized surface, producing a smoother and more even enamel surface. On treating the demineralized surface with nHA-containing universal adhesive, it was observed that a more consistent plane surface with minute cracks throughout the whole surface was noted in 0.5 wt% nHA-containing universal adhesive resin-restored WSLs (Fig. 2e), 1 wt% nHA-containing universal adhesive resin-restored WSLs (Fig. 2f), and 3 wt% nHA-containing universal adhesive resin-restored WSLs (Fig. 2g). These minute cracks decreased gradually with increasing the nHA loading in the universal adhesive resin, from 5 wt% nHA-containing universal adhesive resin-restored WSLs (Fig. 2h), 7 wt% nHA-containing universal adhesive resin-restored WSLs (Fig. 2i), and finally to 10 wt% nHA-containing universal adhesive resin-restored WSLs (Fig. 2j). The nearly complete obliteration of the cracks was observed in micrographs of 7 and 10 wt% nHA in universal adhesive resin, with an observable clustering of nHA spherical particles.

**Fig. 3:**
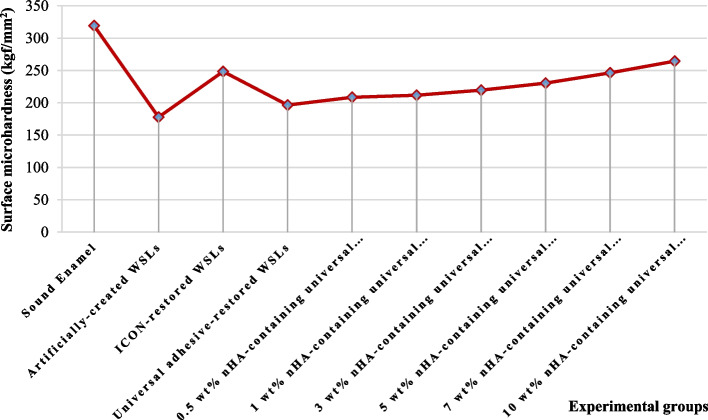
Surface microhardness results of the investigated groups

The means and standard deviations of the surface microhardness (kgf/mm^2^) of the investigated groups are shown in Table 1. The sound enamel (baseline) exhibited the highest mean (319.2 + 3.13), while the artificially-demineralized enamel had the lowest (177.8 + 9.39). Upon the restoration of the demineralized enamel with ICON resin, universal adhesive resin, and nHA-containing universal adhesive resin in ratios of 0.5 wt%, 1 wt%, 3 wt%, 5 wt%, 7 wt%, and 10 wt%, the ANOVA revealed a significant difference among groups (*p* < 0.05). The Tukey test reported that all groups were significantly different from each other at *p* < 0.05. Both the ICON resin-restored group mean (248.3 + 2.82) and that restored with 7 wt% nHA-containing universal adhesive resin (246.32 + 2.72) were significantly different from those of all other groups but were not significantly different from each other. The universal adhesive resin-restored WSLs mean was (196.4 + 6.21), was also significantly different from the mean of the other experimental groups except the one treated with 0.5 wt% nHA-containing universal adhesive resin (208.5 + 8.56). Additionally, no significant difference was recognized among the means of 0.5 wt% nHA-containing universal adhesive resin-restored WSLs (208.5 + 8.56), 1 wt% nHA-containing universal adhesive resin-restored WSLs (211.67 + 6.27), and 3 wt% nHA-containing universal adhesive resin-restored WSLs (219.53 + 3.3). On the other hand, the 5 wt% nHA-containing universal adhesive resin-restored WSLs’ mean (230.39 + 6.9) was significantly different from all other groups’ means; however, no significant difference was detected between it and the 3 wt% nHA-containing universal adhesive resin-restored WSLs’ group mean (219.53 + 3.3). The WSLs restored with 10 wt% nHA-containing universal adhesive resin exhibited a mean value (264.3 + 3.73), which was significantly different from all other groups’ means and represented the most effective formulation in restoring the surface microhardness of the demineralized enamel with a statistically significant difference from the most popular treatment approach using ICON resin. Figure 3 denotes a graphical presentation of the surface microhardness of the experimental groups.

**Table 1:** Means, standard deviations and Tukey’s analysis of the surface microhardness and surface roughness of the studied groups

Group	Surface microhardness (kgf/mm^2^)	Surface roughness (μm)
**I (Sound enamel)**	319.2^a^ **+** 3.13	1^c^ + 0.02
**II (Artificially-created WSLs )**	177.8^g^ **+** 9.39	2.35 ^a^ + 0.21
**III (ICON- restored WSLs)**	248.3^c^ **+** 2.82	1.66 ^b^ + 0.16
**IV (Universal adhesive- restored WSLs)**	196.4^f^ **+** 6.21	1.83^b^ + 0.07
**V (0.5 wt% nHA-containing universal adhesive resin-restored WSLs)**	208.5^ef^ **+** 8.56	0.34 ^d^ + 0.01
**VI (1 wt% nHA-containing universal adhesive resin-restored WSLs)**	211.67^e^ **+** 6.27	0.31 ^d^ + 0.03
**VII (3 wt% nHA-containing universal adhesive resin-restored WSLs)**	219.53^de^ + 3.3	0.26 ^d^ + 0.03
**VIII (5 wt% nHA-containing universal adhesive resin-restored WSLs)**	230.39 ^d^ + 6.9	0.159 ^d^ + 0.01
**IX (7 wt% nHA-containing universal adhesive resin-restored WSLs)**	246.32^c^ + 2.72	0.182 ^d^ + 0.02
**X (10 wt% nHA-containing universal adhesive resin-restored WSLs)**	264.3^b^ + 3.73	0.194 ^d^ + 0.01
***p****-***value**	(*p* < 0.0001)	(*p* < 0.0001)

^*^Means with the same superscript letter in each column are not significantly different at *p* ≤ 0.05

^*^ *nHA* nano-hydroxyapatite, *WSLs *white spot lesions

**Fig. 4:**
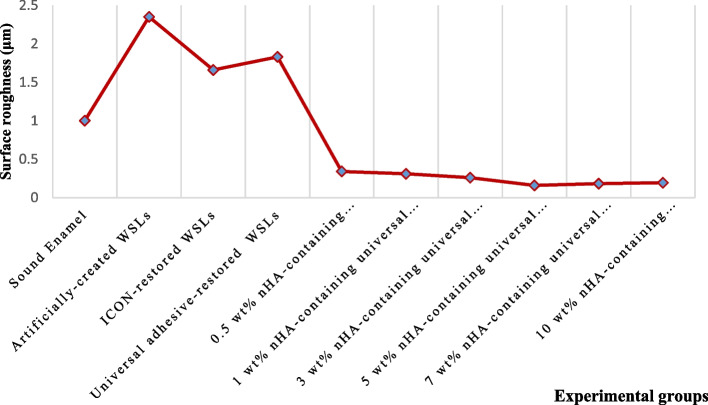
Surface roughness results of the investigated groups

For surface roughness (μm), the results are shown in Table 1. The artificially-created WSLs had the highest mean among groups (2.35 + 0.21) denoting the highest roughness, while the 5 wt% nHA-containing universal adhesive resin-restored WSLs possessed the lowest (0.159 + 0.01). ANOVA revealed a significant difference among the investigated groups (*p* < 0.05). Tukey detected that both means of WSLs restored with ICON (1.66 + 0.16) and universal adhesive resins (1.83 + 0.07) were significantly different from other groups’ means, including sound enamel, artificially-created WSLs, and n-HA-containing universal adhesive resin restored WSLs (*p* < 0.05), though no significant difference was noted between them (the ICON and universal adhesive resin groups). Furthermore, restoring WSLs with 0.5 wt% nHA-containing universal adhesive resin (0.34 + 0.01), 1 wt% nHA-containing universal adhesive resin (0.31 + 0.03), 3 wt% nHA-containing universal adhesive resin (0.26 + 0.03), 5 wt% nHA-containing universal adhesive resin (0.159 + 0.01), 7 wt% nHA-containing universal adhesive resin (0.182 + 0.02), and 10 wt% nHA-containing universal adhesive resin (0.194 + 0.01), showed a statistically significant difference from sound enamel, artificially-created WSLs, and WSLs treated with either ICON resin or universal adhesive resin, nevertheless no significant difference predicted among the nHA-containing universal adhesive resin restored lesions in all investigated ratios. The results of surface roughness are graphically presented in Fig. 4.

**Fig. 5:**
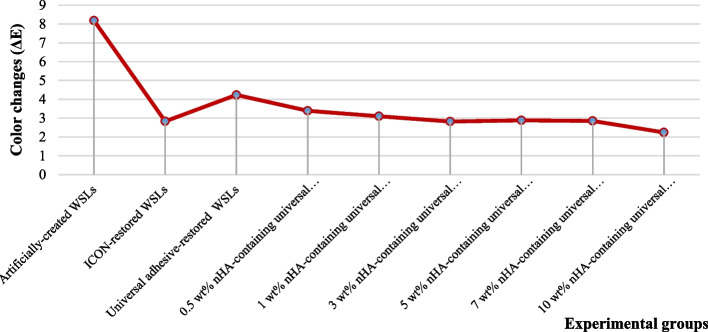
Color changes (ΔE) of WSLs of the different experimental groups

With reference to spectrophotometric analysis, the means and standard deviations of the different treated groups are shown in Table 2 and the results are represented in Fig. 5. The artificially-created WSLs exhibited the highest color difference mean (8.18 + 0.43) from baseline, while WSLs treated with 10 wt% nHA-containing universal adhesive resin restored WSLs had the lowest (2.24 + 0.08). At *p* < 0.05, ANOVA revealed a statistical significance among the various treatment modalities used on the artificially-created WSLs. Tukey test indicated that all groups ΔE means were significantly different from each other and that all treatment approaches could significantly decrease ΔE after treating the artificially-created WSLs (8.18 + 0.43). ICON-treated WSLs’ mean (2.83 + 0.23) was significantly different from the universal adhesive resin-treated group mean (4.23 + 0.26), 0.5 wt% nHA-containing universal adhesive resin-restored WSLs, and 10 wt% nHA-containing universal adhesive resin-restored WSLs (2.24 + 0.08). The universal adhesive resin-treated WSLs group mean was significantly different from both the ICON resin and all nHA-containing universal adhesive resin-restored WSLs groups’ means. Both 1 wt% (3.1 + 0.08) and 5 wt% (2.88 + 0.25) nHA-containing universal adhesive resin-restored WSLs were significantly different from universal adhesive resin-restored WSLs (4.23 + 0.26), and 10 wt% nHA-containing universal adhesive resin-restored WSLs (2.24 + 0.08), while no statistical significance was noted between them and ICON (2.83 + 0.23), 0.5 wt% (3.39 + 0.32), 3 wt% (2.82 + 0.21), and 7 wt% (2.85 + 0.12) nHA-containing universal adhesive resin-restored WSLs.

**Table 2:** Means, standard deviations and Turkey’s analysis of the color change of the studied groups

Groups	Color difference (ΔE)
	Mean **+** SD
II (Artificially-created WSLs specimens)	8.18^a^ **+** 0.43
III (ICON-treated WSLs)	2.83^d^ **+** 0.23
IV (Universal adhesive-treated WSLs)	4.23^b^ **+** 0.26
V (0.5 wt% nHA-containing universal adhesive resin-restored WSLs)	3.39^c^ **+** 0.32
VI (1 wt% nHA-containing universal adhesive resin-restored WSLs)	3.1^cd^ **+** 0.08
VII (3 wt% nHA-containing universal adhesive resin-restored WSLs)	2.82^d^ **+** 0.21
VIII (5 wt% nHA-containing universal adhesive resin-restored WSLs)	2.88 ^cd^ **+** 0.25
IX (7 wt% nHA-containing universal adhesive resin-restored WSLs)	2.85^d^ **+** 0.12
X (10 wt% nHA-containing universal adhesive resin-restored WSLs)	2.24^e^ **+** 0.08
*p*-value	< 0.0001

*Means with the same superscript letter are not significantly different at *p* ≤ 0.05

* *nHA* Nano-hydroxyapatite, *WSLs *White spot lesions

## Discussion

This study was based on the hypothesis of investigating the coadjuvant effect of both nano-hydroxyapatite and universal adhesive resin in enhancing both surface characteristics (in terms of surface microhardness and texture) and aesthetics of white spot lesions. The surface microanalysis and spectrophotometric assessment of WSLs treated with n-HA-containing universal adhesive resin in ratios of 0.5 wt%, 1 wt%, 3 wt%, 5 wt%, 7 wt%, and 10 wt% revealed highly favorable consequences compared to the most popular and effective modern approach employing ICON resin.

All investigated protocols were able to restore the surface microhardness of the artificially-created WSLs, however, 10 wt% nHA-containing universal adhesive resin was the most effective as opposed to ICON resin, either universal adhesive resin alone, or those enclosing 0.5 wt%, 1 wt%, 3 wt%, 5 wt%, or 7 wt% nano-hydroxyapatite. It was also recognized that increasing the nHA ratio gradually from 0.5 wt% up to 10 wt% into the universal adhesive resin resulted in a regular increase in the surface microhardness.

The degree of resin penetration is an important factor that may influence the surface microhardness and should be considered while explaining the results. The resin infltrants have a high penetration coefficient, which enables them to penetrate subsurface lesions more thoroughly, filling the spaces between the remaining enamel crystals of the porous lesion [28]. The universal adhesive alone was not able to infiltrate the demineralized lesion to the same degree as either ICON or that containing nano-hydroxyapatite. For ICON resin, the rigid nature of the aromatic backbone of Bis-GMA (Bisphenol A-glycidyl methacrylate) forming it in conjugation with the strong hydrogen bonds developed by the hydroxyl groups within the structure are responsible for its higher surface microhardness records compared to the universal adhesive resin [29, 30]. Conversely, the flexible aliphatic core of the UDMA (Urethane dimethacrylate) resin, which forms the universal adhesive resin, seems to develop fewer interactions and crosslinks within the porous demineralized enamel than did the ICON resin, revealing lower hardness values [31]. The findings concerning the ability of the resin infiltrants to regain the surface microhardness are consistent with an earlier study [32] reported that the microhardness and surface roughness of the enamels treated with the resin infiltrant were nearly identical to those of healthy enamel, suggesting that this substance would be effective for treating enamel subsurface lesions.

The remaining protein matrix in enamel after demineralization may act as a scaffold for ionic conduction and deposition in the nanogaps of the interprismatic region. Accordingly, on loading the universal adhesive resin by means of nHA with its remineralizing potential, it’s possible that the enamel proteins in the interprismatic area would be able to capture the minerals and permit mineral infiltration along the edges of these crystallites, and this seems to be more prominent with increasing the nHA ratio [33, 34].

The nano-nature of HA enhances the penetration ability of its crystals into the interprismatic spaces of enamel [35]. The resin’s infiltrating nature in combination with the nano-hydroxyaptite remineralizing potentiality, and deposition into the nanogaps explains the higher VHNs observed with increasing the nHA loading in the universal adhesive resin [28, 36–39]. A supportive study [40] tried combining the remineralizing approach with a subsequent resin infiltration and reported significant enhancement of surface microhardness of WSLs. Other supplementary study [41] tried nano-hydroxyapatite-containing toothpaste, and concluded that nHA encourages more minerals to deposit on the outer layer as opposed to the lesion’s body, and this new, highly mineralized surface layer can subsequently inhibit both the diffusion of more mineral ions into the lesion’s deeper sections and the acid diffusion into deeper areas of enamel, besides slowing down demineralization, which would limit enamel recrystallization to the subsurface region. This may strengthen the concept of the treatment approach investigated in our study, which involves loading the nHA within the universal adhesive resin would be better than using nHA alone, particularly that the universal adhesive resin contains 10-MDP which is liable to form a chemical bond with the remaining HA of the tooth structure [25]. As a result, the universal adhesive resin may act by forming a bridge connecting to both the HA of the tooth structure and the nHA contained within the resin, influencing the long-term stability of the coat, yet, however there are no studies to date regarding the issue.

All investigated approaches were efficient in occluding the micropores created by enamel demineralization and developing a smoother surface; however, all nHA-containing universal adhesive resin formulations were able to develop a much smoother surface, even more than sound enamel, and this confirms the ability of these nHA-containing universal adhesive resin to occlude the microcracks within the surface of sound enamel detected by scanning electron microscopy. The penetration liability of the resins greatly influences their ability to infiltrate the porous demineralized enamel, developing a smoother surface [28]. Aswani R et al., confirmed that resin infiltration has strong penetration properties and offers superior surface features, and it can be thought of as a suitable therapy option for restoring early enamel defects [42]. Enan E. et al.*,* also supported the liability of the resins’ infiltration to occlude the pores and develop a much flatter surface [43]. The nHA’s responsibility to improve the penetration potential of the nHA-containing universal adhesive resin infiltrate and to occlude the nanogaps within the porous structure explains the significant reduction in surface irregularity [33-35]. All weight ratios of nHA involvement in the universal adhesive resin had the same capacity for enhancing the surface texture, as confirmed by the insignificant difference among them. A preceding investigation confirmed that a smoother texture of the WSLs was successfully developed using a variety of protocols, including ICON resin, CPP-ACP remineralizing agent, universal adhesive resin, and CPP-ACP combined with universal adhesive resin [40].

Regarding the esthetic restoration of WSLs, both ICON and nHA-containing universal adhesive resin preparations with ratios of 1 wt%, 3 wt%, 5 wt%, 7 wt%, and 10 wt% were able to produce a comparable influence on esthetic. For ICON, the addition of TEGDMA (Triethylene glycol dimethacrylate) to Bis-GMA forming the ICON resin results in a lower viscosity of the resin and better penetration into porous enamel [14]. ICON-dry includes 99% ethanol and is used before ICON resin. By reducing viscosity and contact angle, ethanol helps ICON-dry to penetrate surfaces more effectively. Additionally, longer conditioning by hydrochloric acid gel for 2 minutes erodes the enamel surface layer more effectively than self-etching adhesives [44, 45]. The deeper resin penetration, the higher the approximation of the refractive index of infiltrated enamel (RI = 1.46) to sound enamel (RI = 1.62), and this is consistent with the lower ΔE of ICON resin [46].

On the other hand, the lower penetration of universal adhesive resin governed by the weaker self-etching etching plan seems to be enhanced by coupling it with nHA, lowering the ΔE values and decreasing the refractive index to a value as good as ICON resin-treated WSLs. One relevant study [47] found that nHA toothpaste, as opposed to ICON, did not achieve complete aesthetic improvement due to its infiltration into the surface layer rather than the body of the lesion. Although no studies have yet been conducted to evaluate the ability of nano-hydroxyapatite in conjunction with resin infiltration in the management of WSLs, one recent study [40] attempted a strategy based on a comparable attitude involving a remineralizing approach with subsequent resin infiltration and confirmed their ability to generate better value than the remineralizing agent and the universal adhesive resin infiltrant separately.

This recent short-term study [40] investigated the influence of pairing a remineralizing approach with a minimally invasive one employing resin infiltration while managing WSLs and stated enormously encouraging outcomes in terms of esthetic and surface characteristics, and this study may support these findings. It should be noted that the principal function/purpose of the universal adhesive had been investigated, and its bonding performance to tooth substance, could be influenced in some way by the different nHA loads.

Few studies have been conducted yet that specifically address this strategy; therefore, further collateral investigations, whether short- or long-term ones, that focus on the variables that would influence the degree of mineralization are in demand. Some of these investigations are recommended to think about the presence of 10-MDP in the universal adhesive and its influence on the long-term stability of the esthetic results in the challenging oral environment.

In order to assess the potential, rebound effect of the infiltrated white spots in the long run, it may also be necessary to evaluate this treatment approach after aging, as this may necessitate repeating the treatment application over a certain period.

## Conclusions

The following conclusions could be reached from the data and within the parameters of this study;

- All examined ratios of the nano-hydroxyapatite/universal adhesive formulation resulted in a considerable increase in the surface microhardness of white spot lesions, however, the universal adhesive resin with 10 wt% nHA was the best, even when compared to ICON resin.
- Nano-hydroxyapatite-containing universal adhesive significantly decreased the surface roughness of the porous surface of demineralized enamel to a level less than 0.2 μm.
- Clinically acceptable aesthetics have been achieved with all investigated formulations of nano-hydroxyapatite-containing universal adhesive, with the most favorable effect belonging to the 10 wt% nHA-containing universal adhesive.
- Nano-hydroxyapatite-universal adhesive formulation is a very encouraging contemporary strategy for management of WSLs, coupled with the remineralizing concept and the minimally invasive resin infiltration approach.

## Data Availability

All data presented or analyzed during this study are included in this article.
